# The role of cardiac power and lactate clearance as an indicator of resuscitation success among pediatric patients with shock in the intensive care unit of Cipto Mangunkusumo Hospital

**DOI:** 10.1186/s12887-023-04064-4

**Published:** 2023-05-18

**Authors:** Irene Yuniar, Reni Fitriasari, Yogi Prawira, Setyo Handryastuti, Muzal Kadim, Silvia Triratna, Mulyadi M. Djer

**Affiliations:** 1 Division of Pediatric Emergency and Intensive Care, Department of Child Health, University of Indonesia, Cipto Mangunkusumo Hospital, Jakarta, Indonesia; 2 Division of Pediatric Emergency and Intensive Care, University of Indonesia, Harapan Kita National Cardiovascular Centre, Jakarta, Indonesia; 3 Division of Pediatric Neurology, Department of Child Health, University of Indonesia, Cipto Mangunkusumo Hospital, Jakarta, Indonesia; 4 Division of Pediatric Gastroenterology and Hepatology, Department of Child Health, University of Indonesia, Cipto Mangunkusumo Hospital, Jakarta, Indonesia; 5 Division of Pediatric Emergency and Intensive Care, Department of Child Health, Sriwijaya University, Palembang, Indonesia; 6 Division of Pediatric Cardiology, Department of Child Health, University of Indonesia, Cipto Mangunkusumo Hospital, Jakarta, Indonesia

**Keywords:** *Cardiac power*, *Children*, *Lactate clearance*, *Shock*

## Abstract

**Background:**

Shock in children remains the primary cause of mortality and morbidity worldwide. Furthermore, its management outcome is improved using many hemodynamic parameters, such as cardiac power (CP) and lactate clearance (LC). Cardiac power is a contractility index based on the measurement of flow and pressure, and it is a relatively new hemodynamic parameter with limited studies. In contrast, LC has been proven useful as a target outcome in shock resuscitation. This study aims to explore the values of CP and LC in pediatric shock and their association with clinical outcomes.

**Methods:**

This prospective observational study was conducted on children (1 month-18 years old) with shock at Cipto Mangunkusumo Hospital, Indonesia, from April to October 2021. We measured CP using ultrasonic cardiac output monitoring (USCOM®) and serum lactate levels at 0, 1, 6, and 24 h post-initial resuscitation. Subsequently, the variables were described and analyzed with the resuscitation success, length of stay, and mortality.

**Results:**

A total of 44 children were analyzed. There were 27 (61.4%), 7 (15.9%), 4 (9.1%), 4 (9.1%), and 2 (4.5%) cases of septic, hypovolemic, cardiogenic, distributive, and obstructive shock, respectively. Within the first 24 h post-initial resuscitation, CP and LC had an increasing trend. Compared to children who had successful resuscitation, those who did not have successful resuscitation had similar CP at all time points (*p* > 0.05) and lower LC at 1 and 24 h post-initial resuscitation (*p* < 0.05). Lactate clearance was an acceptable predictor of resuscitation success (area under the curve: 0.795 [95% CI: 0.660–0.931]). An LC of 7.5% had a sensitivity, specificity, positive predictive value, and negative predictive value of 75.00%, 87.5%, 96.43%, and 43.75%, respectively. Lactate clearance in the first hour post-initial resuscitation had a weak correlation (r=-0.362, *p* < 0.05) with hospital length of stay. We found no difference in CP and LC among survivors compared to nonsurvivors.

**Conclusions:**

We found no evidence that CP was associated with resuscitation success, length of stay, or mortality. Meanwhile, higher LC was associated with successful resuscitation and shorter length of stay at the hospital, but not mortality.

## Background

Shock is a circulation failure that creates an imbalance between oxygen delivery and consumption, resulting in global tissue hypoperfusion, decreased venous oxygen content, and metabolic acidosis [1]. Its incidence in children remains a significant cause of morbidity and mortality worldwide, but many undisclosed facts exist on the topic [1, 2]. More than a million shock cases occur annually in the United States [1]. Furthermore, a single-center study in India by Gadappa and Behera reported that shock incidence in a pediatric intensive care unit (PICU) was 8.6% [3]. According to a retrospective study, septic shock was the most frequent type of shock (51%) among children admitted to the emergency department and the PICU, followed by hypovolemic (38.1%), cardiogenic (6.5%), and other distributive shocks (4.4%) [4]. This finding was in accordance with the results from De Backer et al. [5].

The imbalance of oxygen delivery and consumption in the condition of shock has become a risk factor for organ failure and mortality [6]. It is becoming the reason why immediate and accurate hemodynamic support is essential. Many hemodynamic parameters, including invasive and noninvasive methods, are utilized to monitor shock patients. Several minimally invasive and noninvasive parameters were developed to aid in managing shock patients for optimal outcomes, but there is a need for their validation in a certain population [7]. Measuring cardiac output (CO) and its components (preload, afterload, and contractility) provides information about the requirement status of fluid resuscitation, inotropic, or vasopressor drugs. Additionally, it helps diagnose the type of shock, such as hypovolemic, cardiogenic, obstructive, or distributive shock, based on the hemodynamic profile [8].

Among many hemodynamic parameters, cardiac power (CP) has been used as an essential parameter for predicting clinical outcomes in patients with cardiogenic and septic shock. Additionally, it was stated that a low CP is a strong predictor of mortality in patients with shock, especially in cardiogenic and septic shock [9]. Cardiac power is a contractility index calculated based on the principle of fluid derived by flow and pressure divided by 451 [10]. Each global perfusion deterioration that occurs in critically ill patients will be considered to affect cardiac performance and cause hypoperfusion of the myocardium. This topic needs further investigation regarding the CP formula that calculates the CO and mean arterial pressure (MAP), resulting in a more precise and representative CP value calculation of the patient’s general condition [10].

The hemodynamic parameters of macrocirculation and microcirculation assessment during shock management are essential. In contrast, lactate clearance is a microcirculation biomarker that is widely used in clinical practice. For example, it is recommended as a target outcome for resuscitation by the 2020 Surviving Sepsis Campaign [11]. Furthermore, a multicenter observational study conducted by Arnold et al. [12]. confirmed that lactate clearance of less than 10% is a strong predictor of mortality, making this parameter a critical subject to explore as a surrogate marker for microcirculation improvement. In this context, this study aims to evaluate the hemodynamic parameters (cardiac power and lactate clearance) after initial resuscitation and explore their association with resuscitation outcome, length of stay, and mortality.

## Methods

### Study design

This study was a single-center observational study prospectively conducted at the Cipto Mangunkusumo Hospital (CMH), a tertiary-level referral hospital located in Jakarta, Indonesia, from April to October 2021. This study was approved by the Ethics Committee of the Faculty of Medicine Universitas Indonesia-Cipto Mangunkusumo Hospital (KET.406/UN2.FI/ETIK/PPM.00.02/2021, 26th April 2021).

### Patient selection

We included patients aged one month to 18 years admitted with shock and had central venous access consecutively during the study period. We defined shock as blood pressure < 2 standard deviations (SD) based on the normal values for age and/or fulfilling a minimum 3 of these signs: (1) weak peripheral pulse, (2) cold extremity with mottled skin, (3) tachycardia (heart rate > 2 SD based on the normal values for age), or (4) urine output < 1 mL/kg/hour (weight < 30 kg) or < 0.5 ml/kg/hour (weight > 30 kg). The shock was then grouped into five subtypes: septic, hypovolemic, distributive, cardiogenic, and obstructive [2, 13]. We acknowledged that a septic shock is a form of distributive shock. However, in this paper, we would define septic shock as a single subtype due to its common occurrence and different treatment compared to other distributive shocks (e.g., anaphylactic shock and spinal injury). Referring to the 2020 Surviving Sepsis Campaign, we defined septic shock as a severe infection that leads to cardiovascular dysfunction, including low blood pressure, impaired perfusion, and the need for vasoactive drugs [11]. Excluded were those whose parents or guardians had refused to participate and who had comorbidities, such as lethal chromosomal abnormality, cyanotic heart disease, pulmonary hypertension, hepatic disease, or disease of inborn errors of metabolism. We also excluded patients with renal replacement therapy within the first 24 h of admission and death within 24 h post-initial resuscitation.

### Definitions

Cardiac power (W/m^2^) was measured using an ultrasonic cardiac output monitor (USCOM®, New South Wales, Australia) at 0, 1, 6, and 24 h post-initial resuscitation. Cardiac power was calculated using the following formula: [Cardiac Output (CO) x Mean arterial pressure (MAP)]/451 [10]. Similarly, serum lactate and central vein saturation (ScVO_2)_ samples were taken at 0, 1, 6, and 24 h post-initial resuscitation. For lactate and ScvO_2_ examination, we took 2 ml of blood samples from central venous access placed through the superior vena cava, placed it in a tube pre-filled with heparin, and obtained the values through blood gas analysis. Lactate clearance was defined as the proportion of serum lactate change between two time points, calculated by using the following formula: [(initial lactate–final lactate)/initial lactate]x100% [14]. In this study, we define successful resuscitation as ScVO_2_ ≥ 70% and blood pressure > 5th percentile, with a heart rate decrease of > 2 standard deviations (SD) based on the normal heart rate for age [11].

### Outcomes

This study aimed to describe the value of hemodynamic parameters (cardiac power and lactate clearance) in 1, 6, and 24 h after initiation of resuscitation in children admitted with all types of shock. For secondary outcomes, we would examine the association between the hemodynamic parameter (cardiac power and lactate clearance) and clinical outcomes (resuscitation success, length of stay, and mortality).

### Statistical analysis

Based on previous studies in Indonesia, the proportion of septic shock was 51%, while the mean cardiac power and lactate clearance in children with septic shock on day 1 were 0.38 ± 0.35 and 44.17 ± 24.28%, respectively [4, 15, 16]. Therefore, with α of 5% and a margin of error of 15%, the sample size would be 44 children.

Qualitative data were summarized by using proportions (%), while quantitative data were summarized by using mean (SD) or median (min-max). The difference in numeric value would be tested with T-test (parametric) or Mann-Whitney test (non-parametric). We would use the Pearson (parametric) or Spearman rank (non-parametric) test to explore the correlation between numeric variables. Finally, an area under the curve (AUC) would be calculated using receiver operating characteristics (ROC) analysis to see the performance of hemodynamic parameters to predict successful resuscitation. We would also determine the optimal cut-off and calculate the sensitivity, specificity, and predictive values. We defined 0.05 as the limit of statistical significance. The data were analyzed using IBM® SPSS® Statistics version 24 for Windows (IBM Corporation, United States).

## Results

### Subject recruitment and characteristics

**Fig. 1:**
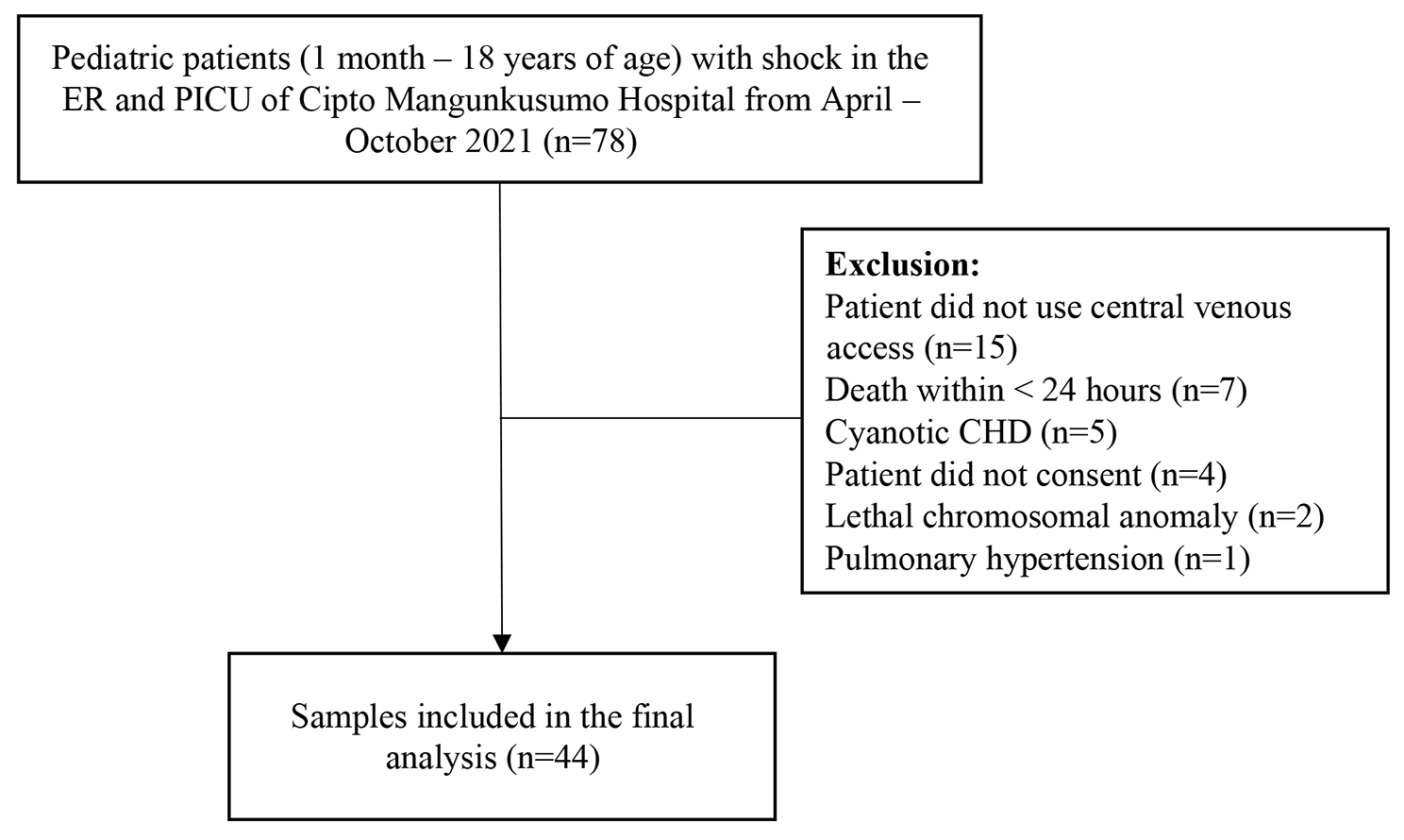
Subject recruitment flow. CHD = Congenital heart disease. ER = Emergency room. PICU = Pediatric Intensive Care Unit

From April–October 2021, there were 78 patients admitted with shock to the emergency room and PICU of Cipto Mangunkusumo Hospital. After the exclusion process, 44 subjects were included in the analysis. The details of patient recruitment flow are summarized in Fig. 1. Most subjects were aged 5–18 years old (54.5%). More patients were admitted due to medical diagnoses compared to surgical diagnoses. Surgical diagnoses were cases that required surgical intervention during the hospital stay, e.g., multiple trauma, tumor, and head injury. Meanwhile, medical cases were diagnoses that did not require surgical intervention, e.g., infection, anaphylaxis, and dehydration. The most frequent shock was septic shock (27 cases, 61.4%), followed by 7 (15.9%), 4 (9.1%), 4 (9.1%), and 2 (4.5%) cases of hypovolemic, cardiogenic, distributive, and obstructive, respectively. The complete patient characteristics are shown in Table 1.

**Table 1:** Characteristics of the subjects

Variable	*n* = 44
Age$, n$	
1 mo – < 1 y	9
$\ge$ 1 y – 5 y	11
$\ge$5 y – 18 y	24
Gender, *n*	
Male	25
Female	19
Diagnoses, *n*	
Medical	38
Surgical	6
Type of shock, *n*	
Sepsis	27
Hypovolemic	7
Cardiogenic	4
Distributive	4
Obstructive	2

mo = month, y = years

### Macrocirculation and microcirculation parameters

Mean arterial pressure and ScvO_2_ increased during 24 h of observation. In this study, the median cardiac power at 1, 6, and 24 h post-initial resuscitation was higher than the initial values (0 h). Among all patients, 29 children (65.91%) presented with a lactate level of ≥2.5 mmol/L. The highest median lactate level was observed at 0 h and decreased at 1, 6, and 24 h after initial resuscitation. Lactate clearance also showed an increasing trend along with the interval of observation. The highest level was observed 24 h post-initial resuscitation, with a median of 36.84%. At 24 h, 17 children (38.64) had persistent lactate level of ≥2.5 mmol/L. The complete macrocirculation and microcirculation parameters are shown in Table 2.

**Table 2:** Macrocirculation and microcirculation parameters

Variable	*n* = 44
Heart rate (beat/minute)	
0h	162.50 (110–197)
1h	141.59 (23.59)
6h	136.61 (21.09)
24h	130.55 (19.42)
Mean arterial pressure (mmHg)	
0h	62.89 (12.06)
1h	67.14 (11.55)
6h	68.07 (13.70)
24h	70.39 (11.75)
ScvO_2_ (%)	
0h	72.86 (17.69)
1h	75.50 (14.12)
6h	74 (34–99)
24h	78.64 (13.30)
Cardiac power (W/m^2^)	
0h	0.37 (0.23–0.77)
1h	0.61 (0.35–0.84)
6h	0.45 (0.25–0.82)
24h	0.55 (0.25–0.81)
Lactate level (mmol/L)	
0h	3.25 (2.20–4.20)
1h	2.30 (1.63–3.50)
6h	2.35 (1.60–3.67)
24h	1.80 (1.23–3.25)
Lactate clearance (%)	
Lactate clearance 0-1h	15.30 ([-108]-[75]
Lactate clearance 0-6h	19.78 ([-150]-[83.61]
Lactate clearance 0-24h	36.84 ([-92]-[87.50]

Values were expressed as mean (SD) or median (min-max).

### Clinical outcomes

Thirty-six (81.8%) subjects had successful resuscitation at 1 and 6 h post-initial resuscitation, and 35 (79%) subjects had successful resuscitation at 24 h post-initial resuscitation. The median length of stay in the PICU and the hospital was 5 (2–8) and 12 (6–21) days, respectively. The mortalities during the first 48 h and the end of the observation were 3 (6.81%) and 16 (36.3%) patients, respectively.

From exploratory analysis, we found no difference in cardiac power at all time points between children who had successful resuscitation and those who did not (ρ > 0.05). However, there was strong evidence that children who had successful resuscitation at 6 h had higher lactate clearance at 1 and 24 h post-initial resuscitation (Table 3).

**Table 3:** The comparison of hemodynamic parameters based on resuscitation success

Hemodynamic Parameters	Successful resuscitation at 6h		*p-*value^*^
	Yes	No	
	*n* = 36	*n* = 8	
**Cardiac power (W/m** ^**2**^ **)**			
1h	0.60 (0.11–1.11)	0.84 (0.11–1.30)	0.140
6h	0.42 (0.14–1.70)	0.76 (1.13–1.10)	0.166
24h	0.53 (0.14-2.00)	0.75 (0.13–1.20)	0.235
**Lactate clearance (%)**			
0-1h	15.30 (2.78–44.92)	10.42 ([-67.78]-[26.27])	**0.013**
0-6h	24.16 (5.6-44.07)	7.14 ([-71.77]-[36.15])	0.166
0-24h	50.0 (-6.89)-(64.28)	-4.68 ([-43.54]-[19.71])	**0.010**

*Mann-Whitney test. Values were expressed as mean (SD) or median (min-max). CP = Cardiac power. h = hour

**Fig. 2:**
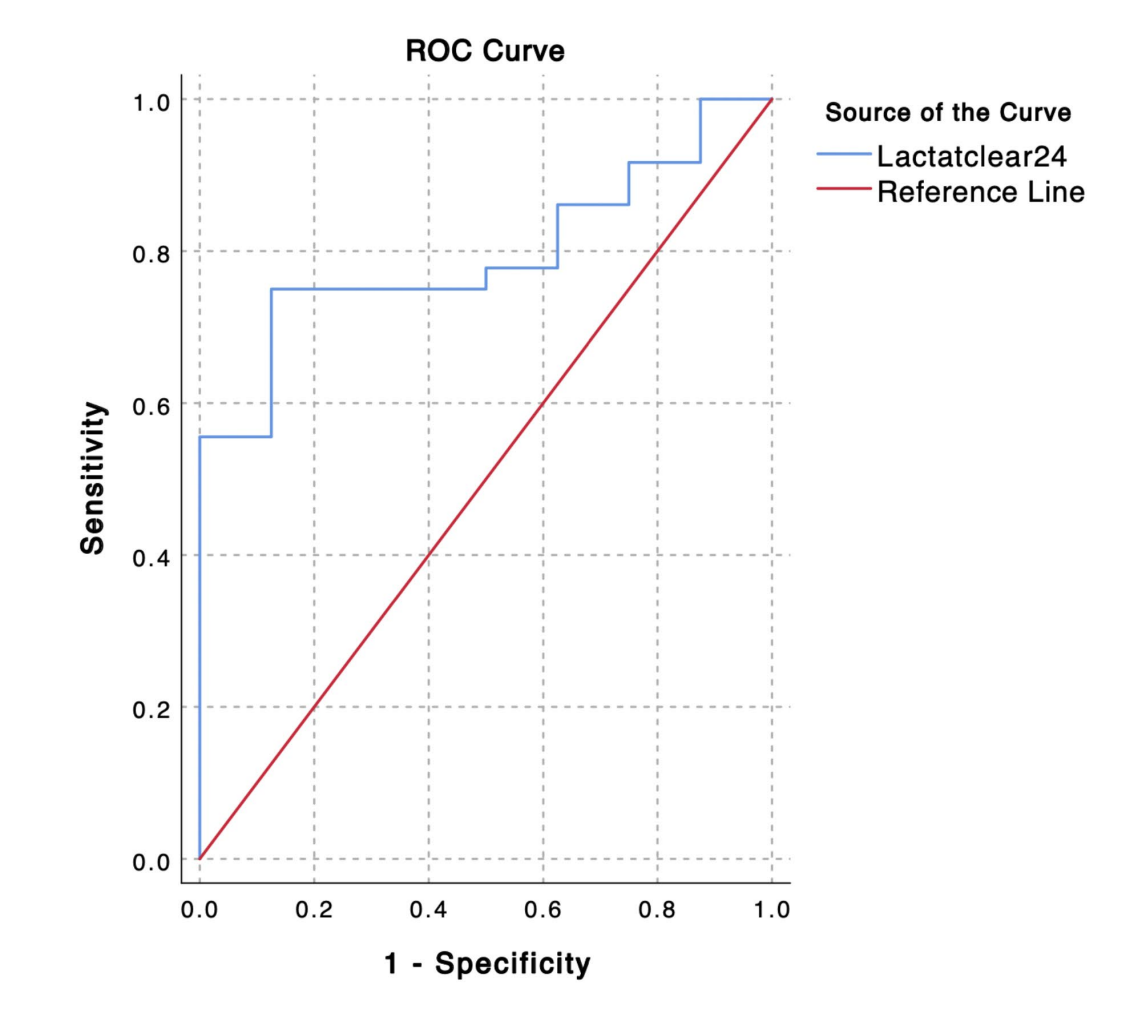
Lactate clearance between 0-24 h was an acceptable predictor of resuscitation success, with an area under the receiver operating characteristic curve of 0.795 (95% CI: 0.660–0.931). ROC = receiver operating characteristic

**Fig. 3:**
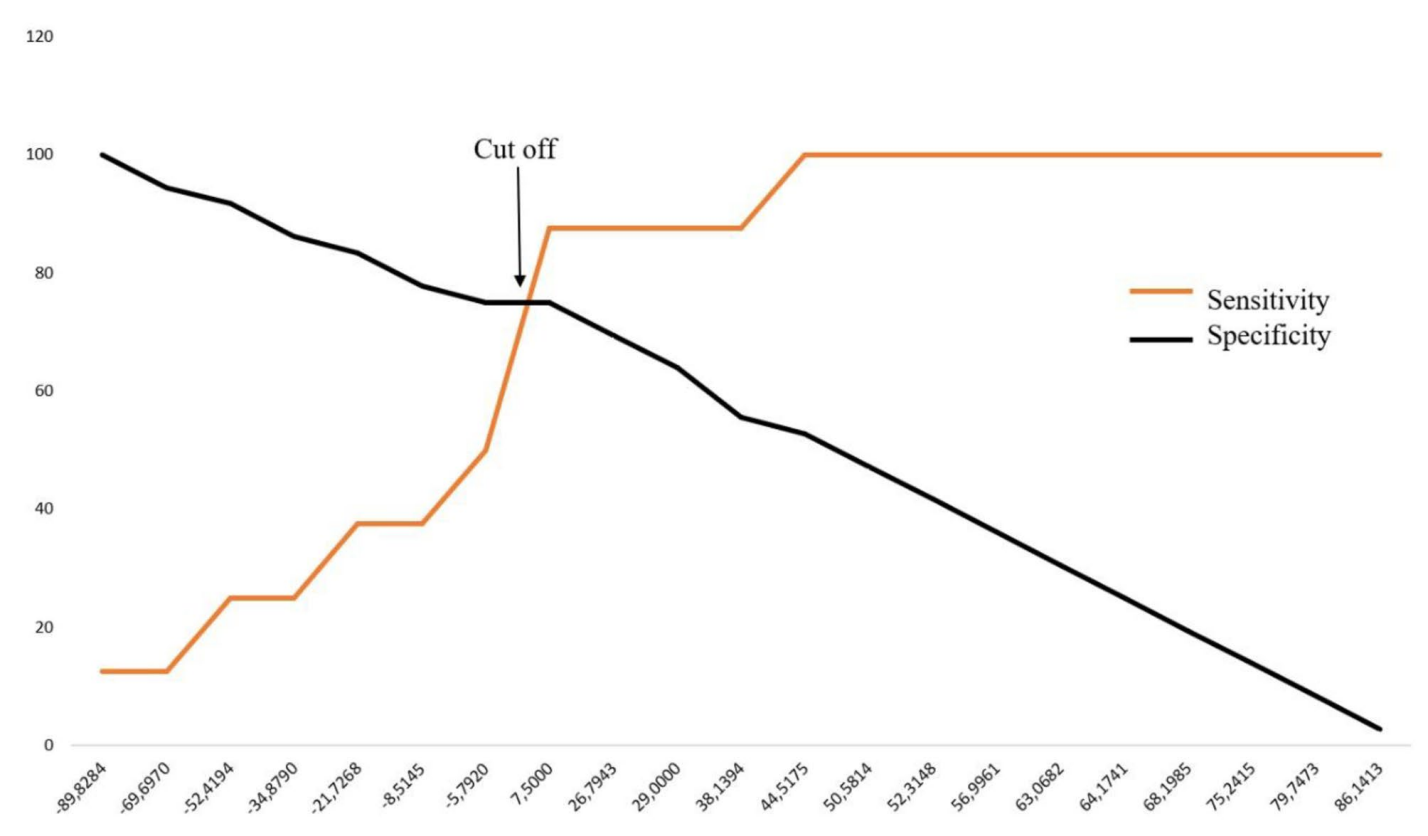
The trade-off between sensitivity and specificity values of lactate clearance to predict resuscitation success at every cut-off. The optimal cut-off for lactate clearance from this graph was 7.5%

The ROC analysis showed that the AUC value of lactate clearance as a predictor of successful shock resuscitation was 95% (95% CI: 57.8–87.88%, p = 0.001) (Fig. 2). The optimal cut-off for lactate clearance to predict resuscitation success in children with shock was 7.5%, with a sensitivity and specificity of 75% (95% CI: 57.80–87.88%) and 87.50% (95% CI: 47.35–99.68%), respectively (Fig. 3). At that cut-off point, the calculated positive and negative predictive values were 96.43% (95% CI: 81.04–99.42%) and 43.75% (95% CI 29.43–59.20%), respectively.

We also did an exploratory analysis to see the correlation between cardiac power and lactate clearance with the length of stay (Table 4). The Spearman rank correlation test revealed strong evidence that higher lactate clearance within the 1st hour after initial resuscitation was correlated with shorter hospital length of stay (r=-0.362, p = 0.017). Finally, we found no difference in cardiac power and lactate clearance between patients who survived and those who did not (Table 5).

**Table 4:** The correlation of CP and lactate clearance to the length of stay in PICU and hospital

Variable	PICU LOS		Hospital LOS	
	**r**	***p-*** **value**	**r**	***p-*** **value**
CP 0 h CP 1 h CP 6 h CP 24 h Lactate clearance 0-1 h Lactate clearance 0-6 h Lactate clearance 0-24 h	0.093 -0.083 0.040 0.248 -0.086 -0.067 0.094	0.578 0.621 0.813 0.133 0.608 0.691 0.576	-0.024 -0.263 0.028 0.112 **-0.362** -0.070 -0.161	0.880 0.089 0.857 0.475 **0.017** 0.656 0.303

*Spearman correlation coefficient. CP = Cardiac power. LOS = Length of stay. PICU = Pediatric intensive care unit

**Table 5:** The comparison of CP and lactate clearance based on mortality

	Mortality at 48 h		*p-*value*	Final mortality		*p-*value*	
Variable	Yes (n = 3)	No (n = 41)		Yes (n = 16)	No (n = 28)		
**Cardiac power**							
0 h	0.90 (0.14–0.99)	0.35 (0.09–0.14)	0.529	0.49 (0.14–1.10)	0.31 (0.09–0.14)	0.090	
1 h	1.13 (0.11–1.20)	0.58 (0.12–1.70)	0.499	0.54 (0.11–1.30)	0.64 (0.12–1.70)	0.807	
6 h	0.73 (0.13–1.10)	0.44 (0.14–1.70)	0.834	0.50 (0.13–1.10)	0.44 (0.14–1.70)	0.442	
24 h	0.69 (0.13–1.05)	0.54 (0.14-2.00)	0.870	0.57 (0.13–1.20)	0.53 (0.14-2.00)	0.421	
**Lactate clearance**							
0-1 h	27.45 (22.73–66.20)	13.63 ([-108]-[75])	0.109	29.85 ([-108]-[66.20])	9.83 ([-70.00]-[75.00])	0.092	
0-6 h	22.72 (0.00-59.15)	18.52 ([-150]-[83.61])	0.895	18.50 ([-150]-[83.61])	39.49 ([-85.71]-[82.61])	0.661	
0-24 h	0.00 ([-92.16]-[39.44])	36.84 ([-87.50]-[87.50])	0.311	14.00 ([-92.16]-[87.50])	46.41 ([-72.73]-[84.78])	0.246	

*Mann-Whitney test. Values were expressed as mean (SD) or median (min-max)

## Discussion

This study explores the value of hemodynamic parameters (cardiac power and lactate clearance) after initial resuscitation in children with shock. This study found that cardiac power and lactate clearance had an increasing trend within 24 h after initiation of resuscitation. Lactate clearance had a good performance in predicting successful resuscitation and a weak negative correlation with length of stay. This study also found no evidence that cardiac power, unlike lactate clearance, could predict successful resuscitation, length of stay, or mortality.

Our samples had a wide age range (1 month to 18 years), with school-aged children and adolescents comprising the largest age group. This finding differs from previous studies where critically ill diseases and shocks were more prevalent in children under five years old, who were more susceptible to infection and at higher risk of developing sepsis shock due to an immature immune response [4, 17]. The male and female subjects were 56.8% and 43.2%, respectively. This is in accordance with the results of Watson et al., which states that the prevalence in males was higher than that in females [18]. Furthermore, the initial diagnosis consisted of 38 (86.64%) medical cases. This is due to the higher proportion of medical cases in the PICU of CMH. Our study also showed that the prevalence of each type of shock was similar to that in previous studies [1, 4], where the most common type was septic shock, with 27 cases (61.4%).

From the hemodynamic parameters in this study, cardiac power was lowest at initial resuscitation and then increased progressively at 1, 6, and 24 h post-initial resuscitation. The cardiac power recorded in our study was similar to previous findings in children with septic shock, which showed an increased trend during the first three days after resuscitation [15]. The result reflected the typical clinical course of shock after initial resuscitation, where CO and MAP were optimized through fluids, vasoactive, and inotropic drugs [11]. On the other hand, the serum lactate level was highest at admission, with a median level of 3.25 mmol/L (2.20–4.20), then decreased steadily. It showed that improved perfusion from resuscitation subsequently made anaerobic metabolism subside and drove lactate out of the body. In this study, lactate clearance was higher with every observation interval, and its highest median was discovered at 24 h post-initial resuscitation. This is similar to the study conducted by Park et al., which stated that lactate clearance was highest in the 24-hour observation period [19].

Then, we found no difference in cardiac power between children who had successful resuscitation compared to those who did not. Despite its proven use to predict in-hospital mortality in patients with cardiogenic shock [10], our study is among the first to explore the value of cardiac power to predict resuscitation success among children with all types of shock. The non-significant difference might be due to the limited sample size of our cohort or the nature of cardiac power itself. Cardiac power is a calculation based on the CO (heart rate and stroke volume) and MAP, which would continuously be improved due to the fluid and drugs during resuscitation. While cardiac power reflected the parameters of macrocirculation, it failed to capture the extent of tissue oxygenation reflected in ScvO_2_, which was included as one of the criteria to determine successful resuscitation in this study [11, 20].

In contrast with cardiac power, we found strong evidence that lactate clearance was higher in children who had successful resuscitation than those who did not. The result was possible because lactate clearance captured the ScvO_2_, a marker of oxygenation and metabolism, better than cardiac power. Furthermore, some studies found that lactate clearance might be as similar as or even more sensitive marker of in-hospital and 28-day mortality compared to ScvO_2_ in adult patients with severe sepsis or septic shock [21, 22]. Despite the scarce data among pediatric patients, lactate clearance is also useful in predicting mortality in adult patients with cardiogenic shock [23]. The lactate trend is indeed recommended by the Surviving Sepsis Campaign 2020 as a target parameter to guide resuscitation due to its association with persistent organ dysfunction and mortality; however, the recommendation did not specify any cut-off as a target [11, 24, 25]. Previous studies in pediatric patients had investigated lactate clearance to predict mortality with different cut-offs and time points [4, 16, 24], but none had explored resuscitation success. Based on our data, a cut-off value of 7.5% was discovered as an optimal 24 h lactate clearance to predict resuscitation success in children with all types of shock, with sensitivity, specificity, and positive predictive values of 75%, 87.5%, and 96.43%, respectively. Thus, we suggest future studies with bigger statistical power to add external validity to this result.

In this study, the median length of stay in the PICU and the hospital was 5 and 12 days, respectively. Cardiac power did not correlate with the length of stay; however, higher lactate clearance during the first hour after initial resuscitation was correlated with a shorter length of stay in the hospital (r= -0.362, *p* = 0.017). Previously, a study by Park et al. showed that patients with higher lactate clearance (≥64% in 24 h) had longer stay in the ICU (8.0 (4.0–16.5) days *versus* 6.0 (2.0–15.0) days, *p* = 0.002) and hospital (14.0 (8.0–28.0) days *versus* 11.0 (5.0–27.0) days, *p =* 0.001) [19]. However, it might be because of the higher in-hospital mortality rate in patients with lower lactate clearance (25.5% *versus* 42.7%, *p* < 0.001). Moreover, a systematic review found that lactate clearance-directed therapy both reduced the length of stay in ICU by two days (95% CI: -3.23 to -0.05 days) and lowered in-hospital mortality by 32% (95% CI: 18–44%) compared to ScvO2 guided therapy in adult patients [26]. All studies included in the review used lactate clearance goals during the first 6 h; therefore, this result opened a possibility regarding the importance of lactate clearance, especially within the first hour after admission, to be explored further.

The 48 h and final mortality rates were 6.81% and 36.3%, respectively. The mortality rate during the first 48 h, closely related to the shock incidence and its subsequent management, was similar to the mortality rate in the world, which were approximately 6–15% [2, 27]. The high mortality rate at the end of the observation period was probably due to multiple factors, one of which was the severity of the underlying disease. Our study reported a similar mortality rate to a previous study in our center, in which the in-hospital mortality was 28.26% (95% CI: 19.36–38.61%) [4].

Among children in our cohort, we found no difference in cardiac power and lactate clearance between survivors and nonsurvivors. Previously, cardiac power was found as the strongest predictor of mortality in patients with cardiogenic shock [10]. However, the study enrolled exclusively adults with cardiogenic shock due to coronary arterial disease. In contrast, in this study, we enrolled children with all types of shock, with septic shock as the predominant type. A cardiogenic shock occurs when the heart fails to pump enough blood into the circulation, while patients with other types of shock (e.g., pure hypovolemic and distributive shock) have entirely different pathophysiology. Septic shock is also different from cardiogenic shock, despite the fact that there might be some degree of cardiac dysfunction in septic shock patients [13]. Cardiac power exclusively represents the heart pumping ability; therefore, the difference in the primary pathophysiology between cardiogenic shock and other types of shock might play a role in this finding.

Previously, a systematic review found that higher lactate clearance was associated with 66% (95% CI: 47–78%) lower risk of all-cause mortality in adults [28]. However, there were some caveats to be considered in interpreting the result, such as the lack of consensus in determining the cut-off used, the heterogenous diagnosis of the patient population, and the difference in initial lactate. Four out of 14 studies had initial lactate of ≥4 mmol/L. A previous study in our hospital found that patients with hyperlactatemia were more likely to have a lactate clearance of ≥10% (*p* = 0.049); however, it failed to prove if lactate clearance was associated with less mortality (31.3% versus 17.6%, p = 0.362). Nevertheless, our findings could not refute the usefulness of lactate clearance as a surrogate marker of microcirculation due to limited samples.

### Limitations

Due to the limited number of samples, this study might lack the power to detect the difference in cardiac power and lactate clearance between the survivors and nonsurvivors. Moreover, our population was heterogenous in terms of diagnosis and age range. This study also did not use the arterial line as the gold standard for measuring MAP and therefore was subject to measurement bias. The cardiac power itself may have a limitation as a parameter in evaluating shock resuscitation because it could not capture the nuances of systolic and diastolic dysfunction that might happen in different types of shock [29]. Moreover, lactate clearance might also be confounded by initial lactate. Therefore, more pediatric studies are needed to elucidate the association of hemodynamic parameters (cardiac power and lactate clearance) and mortality in pediatric shocks, with types of shock and initial lactate included as confounding factors in the regression analysis.

## Conclusions

The value of cardiac power in children with shock were 0.37 (0.23–0.77), 0.61 (0.35–0.84), 0.45 (0.25–0.82), and 0.55 (0.25–0.81) W/m^2^ at 0, 1, 6, and 24 h after initiation of resuscitation, respectively. The lactate clearance had an increasing trend at 1, 6, and 24 h after initiation of resuscitation, with a median of 15.30 ([-108]-[75])%, 19.78 ([-150]-[83.61])%, and 36.84 ([-92]-[87.50])%, respectively.

There was no evidence that cardiac power is associated with resuscitation success, correlated with length of stay, or associated with mortality. In contrast, lactate clearance within 24 h after initiating resuscitation was higher in patients who had successful resuscitation than those who did not. Higher lactate clearance was correlated with a shorter length of stay in the hospital. Future studies may explore the external validation for our findings and further investigate the association of hemodynamic parameters with mortality in pediatric patients with shock.

## Data Availability

The datasets used and/or analyzed during the current study are available from the corresponding author upon reasonable request.

## Abbreviations

AUC: Area under the curve
CHD: Congenital heart disease
CI: Confidence interval
CO: Cardiac output
CP: Cardiac power
EGDT: Early goal-directed therapy
ER: Emergency room
LOS: Length of stay
MAP: Mean arterial pressure
PICU: Pediatric intensive care unit
PPV: Positive predictive value
NPV: Negative predictive value
ROC: Receiver operating characteristic
ROC: receiver operating characteristic
CMH: Cipto Mangunkusumo Hospital
ScvO_2_: Central vein saturation
SD: Standard deviation
USCOM: Ultrasonic cardiac output monitoring.
